# Biocarbon from peanut hulls and their green composites with biobased poly(trimethylene terephthalate) (PTT)

**DOI:** 10.1038/s41598-020-59582-3

**Published:** 2020-02-24

**Authors:** Maisyn Picard, Suman Thakur, Manjusri Misra, Deborah F. Mielewski, Amar K. Mohanty

**Affiliations:** 1 Bioproducts Discovery and Development Centre, Department of Plant Agriculture, University of Guelph, Crop Science Building, 50 Stone Road East, Guelph, Canada; 2 School of Engineering, University of Guelph, Thornbrough Building, 50 Stone Road East, Guelph, Canada; 3 Research and Innovation Center, Ford Motor Company, Dearborn, MI 48124 USA

**Keywords:** Natural product synthesis, Polymer synthesis, Sustainability, Green chemistry, Materials chemistry, Polymer chemistry, Chemistry, Chemical engineering, Characterization and analytical techniques, Environmental, health and safety issues, Engineering, Chemical engineering

## Abstract

There are millions of tons of post-food processing residues discarded annually. Currently, these waste materials are discarded to landfill, used as animal feed or incinerated. This suggests that there are potential uses for these materials in value-added applications. This work focuses on the characterization and valorization of peanut hulls through the generation of green composites. Peanut hulls were pyrolyzed at 500 °C and analyzed to discover their unique surface morphology and relatively low ash content. Raman spectral analysis determined I_D_/I_G_ values of 0.74 for the samples, suggesting greater graphitic content than disordered carbon content. Such results were confirmed in X-ray diffraction analysis by the presence of (002) and (100) planes. Partially biobased engineering thermoplastic, poly(trimethylene terephthalate) (PTT), was combined with 20 wt.% biocarbon. The tensile and flexural moduli improved with the addition of biocarbon, and the bio-content increased from 35 to 48 wt.% as compared to neat PTT. The higher temperature biocarbon was found to have superior performance over the lower temperature sample. The enhanced sustainability of these materials suggested that peanut hulls can be valorized via thermochemical conversion to generate value-added products. Future works could focus on the optimization of these materials for non-structural automotive components or electrical housings.

## Introduction

**Figure 1:**
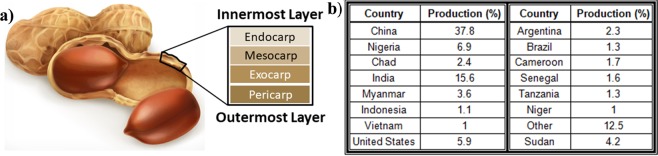
(**a**) schematic representation of peanut structure purchased from iStockphoto.com, (**b**) annual global peanut production^67^.

Biomass is generated from a number of biological sources, including waste from the agricultural industry, as well as beverage and food processing industries. There have been substantial amounts of work to valorize these materials^1^ by extracting compounds^2^, repurposing for value-added products^3^ or burning as energy sources^4^. Peanut hulls are an abundantly available, low-cost and sustainable biomass. PHs are the outer shell which encompasses the nut (Fig. 1a). The largest global producers of peanuts are displayed in Fig. 1b, however, they are grown in many other areas around the world. It was estimated in 2017 that there were 45 million metric tons of peanuts produced worldwide^5^. Since PHs comprise 21–29% of overall peanut weight^6^, at least 9.4 million metric tonnes were produced in 2017.

This study takes a comprehensive look at biomass generated from PHs that were grown in southern Ontario (Canada). PHs consist of four major structural components. These four layers are the pericarp, exocarp, mesocarp and endocarp arranged in order of outermost to innermost layers (Fig. 1a)^7^. Together, these layers contain different ratios of cellulosic materials such as lignin, cellulose and hemicellulose, as well as small amounts of protein and pectin^8^. Peanut hull biomass is abundantly available and to date has had limited use in value-added products. In fact, the PHs generated in Ontario are spread back on the fields after completion of the harvest season. On the global scale, this biomass is readily available in large quantities, obtained at a relatively low cost and is renewed each year. Further examination of current uses is required in addition to the development of value-added products from this biomass.

The work that has been completed to generate sustainable value-added products or find green applications for PHs, since they are biobased and sustainable precursors, is discussed below. In work completed by Brown *et al*., PHs were used successfully as an adsorptive agent for heavy metals in solution. The PHs were able to remove 90% of metal ions in solution in the first 20 minutes of exposure^9^. Composites containing PHs and epoxy have been fabricated to generate value-added and cost effective products^10^. However, PHs have also been modified thermally to extend their use in other applications. Pyrolysis, the thermochemical conversion of biomass in an atmosphere which lacks oxygen^11^, has been used to modify PHs. The process results in the release of volatile constituents of the biomass and in the generation of biochar and bio-oil^12^.

Biochar is the solid product left after biomass pyrolysis and is also referred to as biocarbon^1^. The use of biocarbon is still under extensive review, with the development of novel applications each year^13^. In agronomy, biocarbon has been used as a soil amendment agent that can be spread on soil and increase retention or absorption of different compounds^12^. In the chemical industry, biocarbon is used as a water treatment material and an absorbent as well as a catalyst. More obvious uses of the biocarbon would be in fuel industries where it can he used in combustion processes and gasification as well as co-firing processes^14^. Biocarbon also possesses the ability to absorb materials, including organic compounds and heavy metals. The absorption of materials is fostered by the surface functionality of the biocarbon, such as oxygenated groups, and, therefore, provides a low-cost absorbent material. Similarly, the absorptive abilities fostered the development of biocarbon as a storage material for gases such as carbon dioxide and hydrogen^15^. There has been limited work completed with biocarbon generated from PHs to repurpose the material or generate value added products. Previously, biocarbon generated from PHs pyrolysed at temperatures in the range of 500–1000 °C was used to generate anodes for lithium batteries^16,17^. In work completed by Lv *et al*., some samples were subjected to activation processes, whereas others were tested without modification. However, both samples had relatively good electrochemical properties^17^.

The present work carried out physiochemical, mechanical, thermal and morphological analysis of PHs and biocarbon generated from their pyrolysis. Furthermore, a comparison was made of these properties with regard to other nut shell biomass as a comparative and reference material. Suggestions are made for green and sustainable uses for PHs.

## Materials and Methods

### Preparation of samples

#### Collection of peanut hulls and the grinding process

Peanut hulls (PHs) of the Valencia variety were donated by Picard Foods Partnership. (Waterford, Ontario, Canada). During the shelling process, peanut hulls are cut into pieces nearly 1 cm in diameter. The shells were dried immediately after processing and stored in dry conditions. The samples of PHs received were milled through a 1 mm sieve in a Retsch GmbH Grinding Mill (Haan, Germany) at 6000 rpm. Samples were only milled to a smaller size due to the small size of the pyrolysis vessel available. On a commercial scale, milling would not be required, thus no resources of time and energy would be required. The milled samples were then placed in an 80 °C oven overnight to dry and reach a moisture content of 1% or less. Moisture content was confirmed using an infrared moisture analyzer (Sartorius, Germany).

#### Preparation of biocarbon

**Figure 2:**
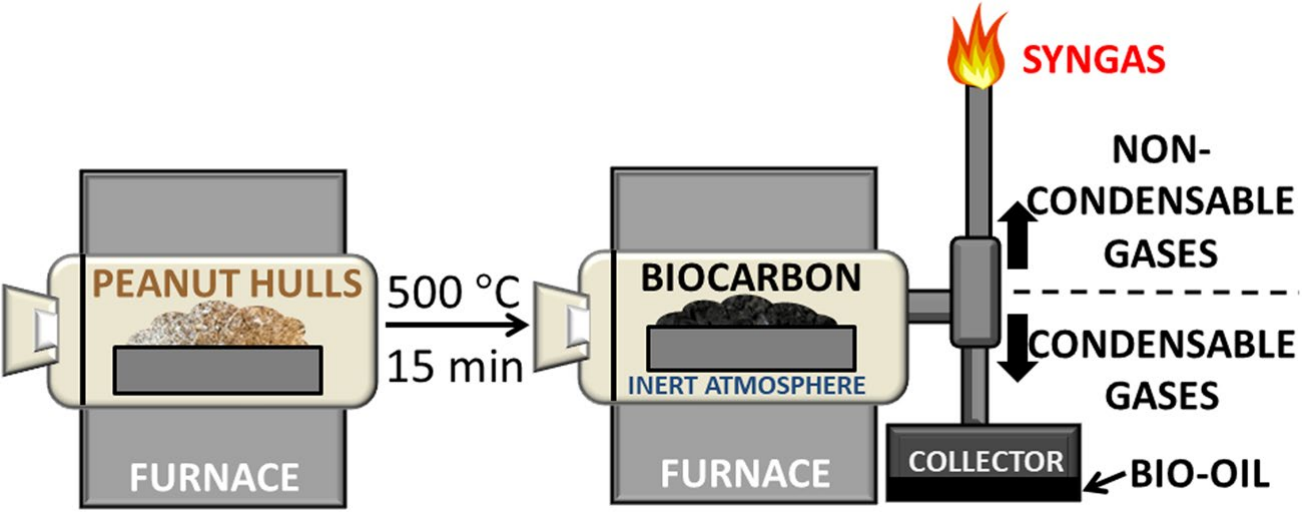
Vertical pyrolysis apparatus for biocarbon synthesis drawn by author.

The biocarbon was placed in a vertical tube pyrolyzer (Carbolite Gero Ltd, UK). Approximately 60 g of ground PH was loaded into the vessel and sealed (Fig. 2). Nitrogen was applied at a rate of about 1.12 SLPM. Samples were heated to 500 °C at 10 °C/min and maintained at 500 °C for 15 min. Once cooled to room temperature, samples were placed in a Fritsch Pulverisette ball mill (Idar-Oberstein, Germany) for 1 hr at 300 rpm. After ball milling, samples were dried to obtain a very low moisture content in preparation for storage.

#### Composite preparation

Composites were prepared using 20 wt.% PH biocarbon in combination with 80 wt.% poly(trimethylene terephthalate) (PTT). PTT, Dupont’s trademark Sorona 3301 BK 001 (Delaware, USA), is a high temperature engineering thermoplastic with 35% biobased content. The composites were made in an Xplor DSM micro injector (Heerlen, Netherlands) with a co-rotating twin screw configuration. Both the biocarbon and PTT were dried for 24 hr in an 80 °C oven prior to processing. Then, the polymer and biocomposites were processed at 250 °C and 100 rpm with 2 min mix time and a mold temperature of 40 °C. Samples were made for tensile, flexural and impact testing.

### Characterization of biocarbon

#### Physiological analysis

PHs and biocarbon samples were subjected to various types of physiochemical analysis. The elemental composition was obtained from a Thermo Fisher Scientific CHNS Elemental Analyzer (Waltham, USA) which measured the carbon, hydrogen, nitrogen and sulfur contents. Three samples of each material were tested and the results are the mean values. The moisture content was required for elemental analysis and ash content. This was obtained by placing approximately 0.5 g of sample in a Sartorius Moisture Analyzer (Gottingen, Germany) which used infrared to measure the moisture content.

#### Thermogravimetric analysis

The thermogravimetric analysis (TGA) was performed on a TA Instruments Q500 (USA) to determine the thermal degradation characteristics of the materials. The tests were performed in ramp style under a nitrogen environment with a heating rate of 10 °C/min to a final temperature of 800 °C. A secondary TGA analysis was performed in combination with Fourier Transform Infrared Spectroscopy (FTIR) and it is referred to as TGA-FTIR analysis, completed on a TGA5500 machine from TA Instruments (USA) in combination with the FTIR machine discussed below. The experiment was completed in a nitrogen environment, where about 10 mg of PHs were heated to 550 °C at a rate of 20 °C/min. Scans of the sample were completed in intervals of 8 s. The analyzed gases that are produced during pyrolysis can be further discussed in relation to their environmental impact. A Gram-Schmidt curve was produced at maximum degradation temperature, and then all volatile materials were plotted as a comparison to temperature around this point. This generates a relative comparison for the evolved gases during the pyrolysis process. This is referred to as relative absorbance below and was determined from taking the maximum peak height and dividing it into the other volatile component absorbances. Further studies could determine the environmental impact from these gases as a comparison for the feasibility of the material.

#### Ash analysis

A Thermo Fisher Scientific Lindberg Blue M vacuum oven (Waltham, USA) was used to determine ash content of PHs. The test was performed in accordance with ASTM E1755-07. This process requires the samples to be heated to 575 °C with a tolerance of 25 °C.

For the biocarbon, two methods to determine the ash content were used. The same process as in literature^18^ was followed where ash content was determined from a furnace method as well as TGA analysis. The first method, using a furnace, was completed in accordance with ASTM E1762. The second method to determine ash content of PH biocarbon was performed on a TA instruments Q500 (New Castle, USA) in accordance with ASTM E1131-08. This is the same equipment as used for TGA analysis.

#### Conductivity

The electrical conductivity was measured on 1 cm diameter packed PH biocarbon powder samples placed between pistons with a 1 kg load. The resistance of the sample was measured with an Autolab PGSTAT302N FRA potentiostatic module and an AUT85394 Differental Electrometer-Amplifier. This set up was operated under software from Metrohm Autolab B.V (Utrecht, Netherlands). The conductivity was calculated from the measured resistance. The electrical and thermal conductivity methods were completed in accordance with previous literature^18^.

The thermal properties, including: thermal conductivity, specific heat of the biocarbon, and diffusivity were measured with a hot disc placed on a powder column. The experimental setup was a Thermtest Hot Disk TPS 500 (Frederickton, Canada). A hollow steel cylinder was loaded with a sensor and biocarbon between steel pistons. The test conditions were 120 mW heating for 80 seconds.

#### Surface area analysis: brunauer-emmett-teller

Brunauer-Emmett-Teller (BET) analysis was performed to determine surface area of the biocarbon in a nitrogen environment. It is important to note that the outcome of this test is influenced by the ball milling conditions (noted above) in addition to the pyrolysis conditions. Nonetheless, BET analysis was performed with a Quantachrome BET Instrument (Florida, USA) where a sample of approximately 20 mg was outgassed for 3 h at 300 °C. Analysis of the samples, completed via Autosorb-iQ (Florida, USA), was performed by selecting 5 points from the linear region of the volume versus relative pressure graph. The average of two samples was taken.

#### Spectroscopy

The biocarbon and PHs were also subjected to FTIR with 128 scans on a Nicolet 6700 FTIR machine made by Thermo Fisher Scientific (Waltham, USA). The scans captured functional groups within the range of 400 to 4000 cm^−1^ with a resolution of 4 cm^−1^ over a diamond shaped window. Further spectroscopic analysis was completed with a DXR2 Raman Microscope from Thermo Scientific (Waltham, USA). Powdered samples were subject to a 1 mW power laser (532 nm) with 50 times lens under a 50 μm slit. The Raman spectrum of biocarbon was obtained between 800 and 2000 cm^−1^.

#### Scanning electron microscopy

A Phemon ProX Scanning Electron Microscope (SEM) from Phenom-World BV (Eindhoven, Netherlands) was used to collect back scattering electrons on micrographs at 15 kV with zoom up to 10 000×. The SEM was used for morphological analysis of PH biocarbon and also the fracture surface of composites.

#### X-ray diffraction analysis

The crystalline structure of the PH biocarbon was analyzed via Rigaku Multiflex Powder x-ray diffraction (XRD) spectrometer (Tokyo, Japan). At a wavelength of 1.54 Å in a copper x-ray tube (Cu-Kα), the spectrometer was operated at 44 mA and 40 kV. A scan rate of 0.2 °/min was taken of the sample taken over the range (2 $\,\theta$) of 5 ° to 80 °. The peaks were analyzed via Jade 9 software (MDI, Livermore, USA). The domain size of the crystal can be calculated using the Scherrer’s Equation^19^:

$$\xi =\frac{0.9\,\lambda }{FWMH\,\times \,\cos (\theta )}$$

where *ξ* is the domain size (or crystal thickness), *λ* is the x-ray wavelength, FWHM is the full width at half maximum and *θ* is the diffraction angle. To determine the interplanar spacing, the Bragg’s law was used with n = 1, the other variables are the same as above.

$$d=\frac{n\,\lambda }{2\,\sin (\theta )}$$

The stacking height (L_c_) and lateral crystallite size (L_a_) were calculated using the following equations from literature^19^:

$$\begin{array}{c}{L}_{c}=\frac{0.89\lambda }{FWH{M}_{(002)}cos{\theta }_{(002)}}\\ {L}_{a}=\frac{1.84\lambda }{FWH{M}_{(100)}cos{\theta }_{(100)}}\end{array}$$

### Characterization of biocomposites

#### Mechanical testing

An Instron 3382 Universal Test Machine (Massachusetts, USA) was used to perform tensile and flexural tests in accordance with ASTM Standards D638 and D790, respectfully. Tensile samples were placed in pneumatic grips then tested at a rate of 5 mm/min to break within the time (30 s to 5 mins) as specified within the ASTM standard. The flexural samples were tested with a 52 mm span and a crosshead speed of 14 mm/min. Impact testing was performed in accordance with ASTM D256 with a Zwick/ Roell HP25 impact tester (Ulm, Germany). The notched Izod impact samples were tested with a hammer capacity of 2.75 J.

## Results and Discussion

A complete analysis of the PHs and PH biocarbon can be found below. The yield of the 500 °C PH biocarbon was approximately 35% which was similar to other low temperature pyrolysis yields of peanut hulls^20^. Particle size of the original biomass can play a crucial role on the resulting biocarbon^21^ and impact the relative concentrations of evolved volatiles during the pyrolysis process^22^. This should be considered when scaling this process. To combat size related concerns, the biocarbon in this study was ball milled to increase uniformity within the sample.

### Physicochemical, thermal and electrical properties of peanut hull biocarbon

According to literature, the average cellulose, lignin, fiber and protein contents for PHs are 38%, 29%, 65% and 7%, respectively^23^. A comparison of cellulosic and lignin content in different nut shells is tabulated in Table 1. Peanut hulls contained similar cellulose content to that of hazelnut and walnut shells, whereas almond shells possessed a significantly greater cellulose content^24^. However, the lignin content contained in PHs was similar to almond shells rather than hazelnut or walnut shells^24^. The lignocellulose content of a biomass provides essential information for determining if the material can be used in thermochemical, chemical, biochemical^25^ or mechanical applications^26^. Thermochemical applications include combustion for fuel sources/biorefining or pyrolysis, whereas chemical applications require the extraction of valuable compounds contained within the biomass^25,27^. Within the chemical section, biochemical applications may require anaerobic or aerobic digestion of materials for biogas^25^. Lastly, biocarbon may be used in material design with specific emphasis on the mechanical performance. For example, biocarbon (or biomass) can be used as a fibrous filler material (dispersive phase) in combination with a continuous polymeric matrix^28^.

**Table 1:** Cellulosic and lignin content in different nut shells.

	Cellulose (%)	Lignin (%)	Hemicellulose (%)	Source
Peanut hulls (PHs)	34–45	27–33	—	^9,23^
PHs	37	28.8	—	^68^
Hazelnut Shells	26.8	42.9	30.4	^24^
Walnut Shells	25.6	52.3	22.1	^24^
Almond Shells	50.7	20.4	28.9	^24^

#### Physiological properties

The elemental analysis of PHs and other raw nut shells is shown in Table 2. It was found that the PHs used in this experiment contained more carbon and nitrogen but less hydrogen and sulfur than reported elemental composition of PHs by *Perea-Moreno et al*.^4^. The differences could probably be attributed to differences in peanut variety and soil in which the plants were grown. Although all peanuts require sandy soil to grow, the growth of the nuts is also greatly affected by temperature, precipitation and length of the growing season. The peanuts used in this study were grown on Fox soils which are a type of loamy sand with good drainage characteristics^29^. The soil characteristics will impact the composition of the samples. Furthermore, the PHs were from shelled Valencia style peanuts grown in Southern Ontario, identified by their smaller size.

**Table 2:** Elemental compositions of nut shells.

	Weight Percent (wt. %)				Source
	C	N	H	S	
Peanut hulls (PHs)	53.54 ± 5.2	1.28 ± 0.14	5.53 ± 0.08	0.01 ± 0.02	Exp.
PHs	46.42 ± 0.01	0.50 ± 0.01	6.61 ± 0.02	0.55 ± 0.01	^4^
Cashew Shells	49.9	0.7	6	<0.1	^33^
Pistachio Nut Shell	47.9–49.2	0.4–0.9	6.7–7.0	—	^69^
Walnut Shells	47.67	0.34	5.67	—	^70^
Pecan Shells	51.6	0.3	5.7	0.02	^12^
Almond Shells	45.6	<0.5	6.2	<0.05	^30^

It is important to quantify the elemental composition of nut shell agro-waste to analyze potential industrial uses or value-added product development, as well as provide insight into the quality and properties of the biomass^4^. The carbon, hydrogen and oxygen contents of the biomass can be used to determine the calorific value for applications such as bio-energy^30^.

The elemental composition for PH biocarbon and other sources of nut shell biocarbon is given in Table 3. The PHs used in this study contained approximately 73% carbon, 0.83% nitrogen and 1.9% hydrogen. The elemental composition trend of the PH biocarbon in this study was similar to other PH sources where carbon was the major constituent, followed by hydrogen and nitrogen^31,32^ from CHNS analysis. There are small differences in carbon, nitrogen and hydrogen content of nut shells, regardless of the type of nut or pyrolysis temperature^12,30,33^.

**Table 3:** Elemental composition of biocarbon from nut shells.

	Weight Percent (wt. %)				Pyrolysis Temp.	Ref.
	C	N	H	S		
Peanut hull (PH) biocarbon	73.44 ± 13.9	0.83 ± 0.14	1.97 ± 0.11	0	500 °C	Exp.
PH biocarbon	68.27	1.91	3.85	0.09	300 °C	^31^
PH biocarbon	83.76	1.14	1.75	0	700 °C	^31^
PH biocarbon	~78	~2	~9	0	500 °C	^32^
Cashew shell biocarbon	79.2	0.2	1.7	0.06	425 °C	^33^
Pecan shell biocarbon	64.5	0.26	5.3	0.01	350 °C	^12^
Pecan shell biocarbon	61.2	0.51	1.5	0.01	700 °C	^12^
Almond shell biocarbon	71.8	0.45	3.9	0.04	600 °C	^30^

The particle size distribution for the biocarbon particles is shown in Table 4. Particle size analysis determined that 80% of the particles were less than 212 μm in diameter. Analysis of particle size distribution is important to determine the behaviour of the materials as well as to determine appropriate applications. The size of the biocarbon particles relates to the exposed surface area as well as to pore size and volume. For applications in water adsorption, it is essential to have high surface area, pore size and pore volume to maximize water uptake^34^. However, for polymeric composite applications a smaller particle is preferred for enhanced mechanical properties^35^. In fact, Nagarajan *et al*. determined that impact strength was greatest for samples with biocarbon sized between 20–75 μm^36^. The smaller biocarbon particles used in biocomposites will result in larger specific surface area and increased impact strength^37^. This is a direct result of improved stress transfer within the composite material. Furthermore, research has found that smaller sized biocarbon (ball milled for longer duration) reduces the coefficient of linear thermal expansion^37^. This is a very important quality when implementing biocarbon biocomposites into industry and the injection molded samples need to maintain dimensional accuracy. Therefore, the size of this material is highly influential on the materials properties and must be considered if implemented commercially.

**Table 4:** Particle size distribution by mass percent for biocarbon.

**Size**	>1 mm	1 mm- 500 μm	500–300 μm	300–212 μm
Biocarbon particles (wt.%)	2.2	6.7	4.4	6.7
**Size**	**212–125 μm**	**125–75 μm**	**<75 μm**	
Biocarbon particles (wt.%)	35.6	22.8	21.7	

#### Scanning electron microscopy (SEM)

**Figure 3:**
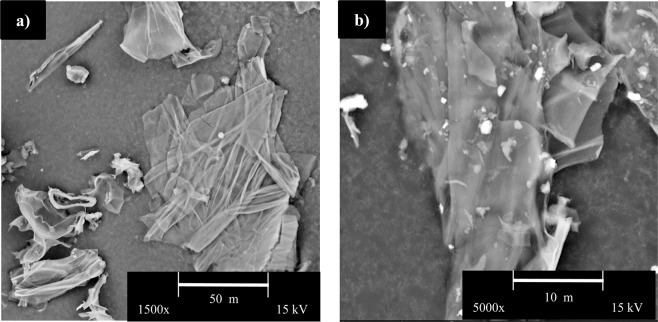
SEM images (**a**) peanut hulls and (**b**) 500 °C peanut hull biocarbon.

SEM images of milled PHs and PH biocarbon are shown in Fig. 3. The PHs displayed fine sheet-like structures with limited pores. Similar findings were reported by Zhong *et al*. during their SEM analysis of PHs^38^. Some morphological features were maintained after pyrolysis. The morphology of PH biocarbon was found have a similar sheet-like structure to PH (Fig. 3b). The morphology of PHs biocarbon also had similarity to that of pecans in worked completed by Zazycki *et al*., as well as to coffee chaff in works completed by Quosai *et al*. Both pecan and peanut shell samples contain structures such as cavities and grooves which aid the absorbance of chemicals (such as dyes) into the biomass^39^, suggesting use in absorbent applications^23^. PHs, similar to coffee chaff, also possessed agglomeration of particles in biocarbon samples^18^. The sheet like structure in the biocarbon is likely to have resulted to increases graphitic content, as noted from the Raman and XRD analyzes below, as compared to other biomasses. Based on the sheet-like structure noted from morphological analysis of both the PHs and biocarbon, it is suggested that the materials may well have potential in green composite applications. There has been significant effort over the last few years to produce sustainable polymeric materials from renewable and green sources^35,40^.

#### Ash content

The ash content of the PH and PH biocarbon samples was determined via two methods for comparison, as done in literature^18^. The ash content for PHs and other nut shells is given in Table 5. It was determined that the ash content for PHs in this study was 4.06% and 2.16% by furnace and TGA methods, respectively. As noted by Suliman *et al*.,. a difference in the temperature of the experiment can impact on the ash content of samples^41^. The variation in temperature was based on differences in experimental procedure. However, the average ash content for all PH samples, as noted in literature and found experimentally, was 3.95%^4,42,43^. Thus, the furnace method with ASTM E1755 provided results nearer to those in literature. In general, the ash content of PHs falls within the range of other nut shells, such as 0.69% and 5.3% for walnut and cashew shells, respectively. In is known that the ash content of the samples is strongly dependent on the nature of the biomass^18^, which in this case was woody biomass. This was confirmed in literature where the average ash content of woody plants ranges from 0.3–7%^18^.

**Table 5:** Ash content for nuts shells.

Nut shells	ASTM	Ash Content	Source
Peanut hulls (PHs)	E1755	4.06 ± 1.55	Exp.
PH	D1102	5.49	^42^
PH	—	3.8	^43^
PH	EN14775	4.26	^4^
Cashew Shells	E1755	5.3	^33^
Pistachio Nutshells	3174–04	2–3.6	^69^
Walnut Shells	—	0.69	^70^
Pecan Shells	D3174	1.6	^12^

The ash content of biocarbon from PHs and other nuts shells is given in Table 6. It was found in this work that the ash content of biocarbon from PHs was similar regardless of the method. The average ash content of PHs, from experiment and cited works, was 4.6%. Therefore, it was confirmed that the ash analysis in this study resulted in a good representation of ash content in PH biocarbon. As with PH ash content, the PH biocarbon ash content was between that of pecan shells (at 350 °C)^12^ and almond shells^30^.

**Table 6:** Ash content for biocarbon derived from nut shells.

Biocarbon	ASTM	Pyrolysis Temperature (°C)	Ash Content	Source
Peanut hull (PH) Biocarbon	E1131	500 °C	4.39 ± 1.55	Exp.
PH Biocarbon	E1762	500 °C	4.82 ± 1.14	Exp.
PH Biocarbon	—	300 °C	1.24 ± 0.08	^31^
PH Biocarbon	—	700 °C	8.91 ± 0.08	^31^
Pecan Shell Biocarbon	D3174	350 °C	2.4	^12^
Pecan Shell Biocarbon	D3174	700 °C	5.2	^12^
Almond Shell Biocarbon	—	600 °C	6.4	^30^

#### Thermogravimetric analysis

**Figure 4:**
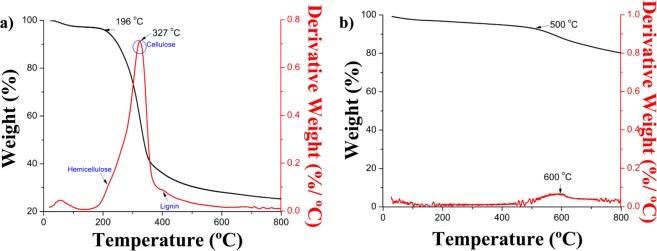
Thermogravimetric analysis of (**a**) peanut hulls and (**b**) 500 °C peanut hull biocarbon.

The thermogravimetric analysis (TGA) was performed to investigate the decomposition of PHs and further test the thermal stability of biomass (Fig. 4a). The initial 5% loss in weight is noted as the slight downward slope of the curve, and was attributed to the moisture content in the samples, as noted in works by Moreira *et al*. with cashew shells^33^. The peanut hulls were able to maintain relatively stable until nearly 200 °C, at which point the weight percent curve begins a transition to a much steeper curve. The maximal degradation peak for PHs was 327 °C. Peanut hulls could not be used for composite applications with high temperature plastics as the material would degrade with the processing temperatures that are often greater than 200 °C. This is common with many food industry waste natural fillers such as apple^3^ and grape^28^ pomace. The derivative weight curve of peanut hulls was used to identify key constituents of the biomass during the degradation process. On closer analysis of this curve, it was noted that there were three characteristic peaks at 223 °C, 327 °C and 402 °C accounting for the decomposition of hemicellulose, cellulose and lignin, respectively^18,28^.

The PH biocarbon thermogravimetric analysis (Fig. 4b) shows a substantial increase in thermal stability. In fact, the sample did not begin to degrade until 500 °C. The stability is maintained because the pyrolysis at 500 °C would have already removed any volatile matter. The sample begins to experience some degradation at 600 °C. The exceptional thermal stability of this material suggests its use in high temperature composites or electrical applications such as supercapacitors.

#### Fourier transform infrared spectroscopic analysis

**Figure 5:**
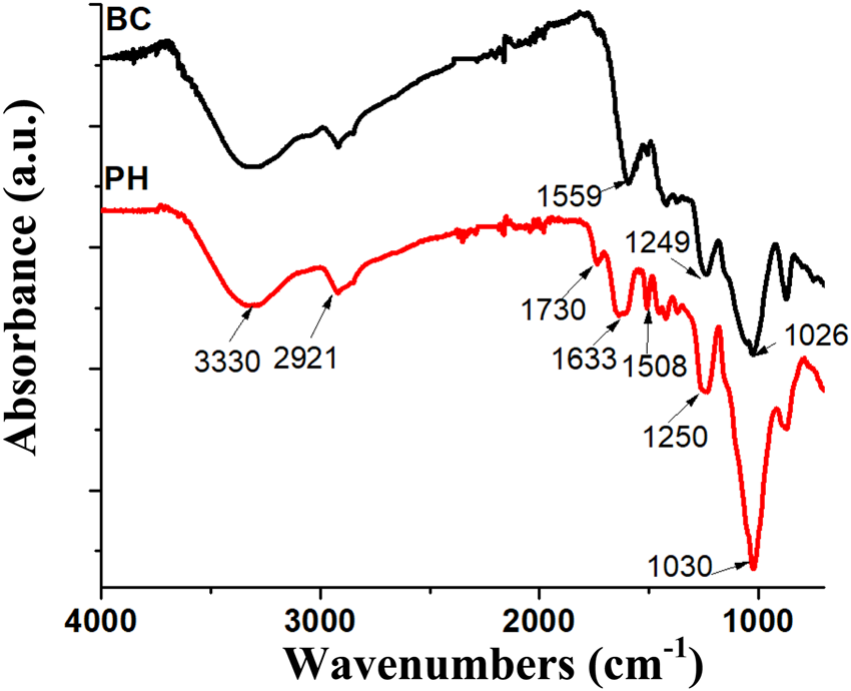
FTIR spectra for peanut hulls (PH) and 500 °C peanut hull biocarbon (BC).

The Fourier transform infrared (FTIR) spectrum for the PHs (Fig. 5) contains broad peaks at 3330 cm^−1^ and 1030 cm^−1^ as well as prominent peaks at 2921, 1730, 1633, 1508 and 1250 cm^−1^. The broad peak noted by the maximal value at 3330 cm^−1^ was also found in the FTIR of peanuts shells in literature. According to Lui *et al*., the broad peak within this region is attributed to the stretching vibrations of O-H^44^. Furthermore, the broad peak at 1030 cm^−1^ was found to be due to the present of cellulose and hemicellulose C-O-H stretching^44^. The peak at 2921 cm^−1^ in PHs was indicative of the C-H stretching of methyl and methylene groups found in the lignocellulosic constituents. Similarly, the peak at 1730 cm^−1^ is probably a result of stretching of C=O groups of hemicellulose and cellulose^42^. Furthermore, PHs naturally contain lignin, as noted by the aromatic conjugated carbonyl C=O stretching vibrations at 1633 cm^−1^ and also C=C stretching of the aromatic rings at 1508 cm^−1^ ^42^. Again, the presence of lignin is confirmed at 1250 cm^−1^ where the C-O stretching vibrations were noted. The presence of lignocellulosic, such as cellulose and lignin materials, was confirmed by literature in Table 1, and confirmed to be present in the PH samples used in this study by FTIR analysis.

The FTIR spectrum for biocarbon contained many similar peaks to those of the PH (Fig. 5). However, there was a decrease in intensity of the peaks and the loss of some volatile matter removed some peaks^39^. The major peaks on the biocarbon spectra were located at 3300 cm^−1^, 1559 cm^−1^, 1249 cm^−1^ and 1026 cm^−1^. The peaks at 3300 cm^−1^ and 1026 cm^−1^ were also found in biocarbon derived from pecans. The peak at 1559 cm^−1^ was also found in woody biomass samples pyrolyzed at 500 °C^34^ which, according to literature, can be attributed to vibrations of aromatic C=C as well as C=O stretching.

#### TGA-FTIR analysis

**Figure 6:**
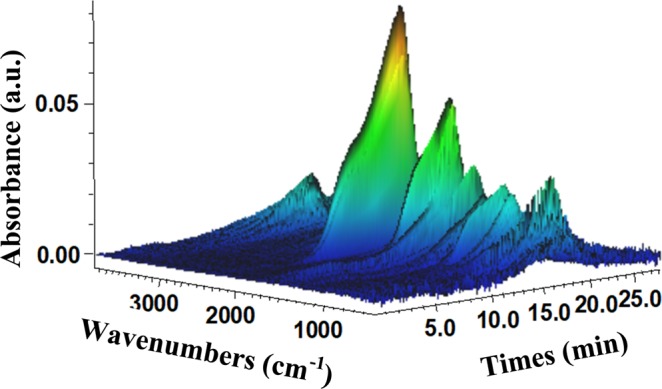
TGA-FTIR of peanut hulls heated to 550 °C.

The TGA-FTIR analysis is important to determine the volatile materials produced during the heating process. The information from this analysis can be further studied to determine the environmental impacts of pyrolysis of biomass^45^. Almost all volatile components were released between 10 and 20 minutes of heating. The major components released from these samples are displayed in Fig. 6 and further described in Table 7.

**Table 7:** TGA-FTIR analysis of peanut hulls during heating.

Species	H_2_O	Hydrocarbons	CO_2_		CO	Carbonyl	Ether
Wavenumbers (cm^−1^)^47^	3400–4000	2700–3000	2250–2400	586–726	2000–2250	1650–1900	1000–1450
Functional groups^47^	O-H	C-H	C=O	C=O	C-O	C=O	C-O, C-C
Vibration type^47^	Stretch	Stretch	Stretch	Bend	Stretch	Stretch	Stretch
Relative absorbance	0.33	0.14	1.00	0.34	0.08	0.63	0.35

The maximal degradation temperature was 366 °C for this analysis and it occurred at almost 18 minutes. The wavelengths corresponding to different volatile materials have been discussed in literature and are included in Table 7 in relation to their wavenumbers, functional groups^46^ and vibration type^47^. The Gram-Schmidt, found in the Supplementary Information (Fig. S1), was used to determine the relative absorbance of the volatile matter. As with other biomasses the major product is carbon dioxide^47^. The production of this gas should be taken into account for further implementation of the materials. As mentioned previously, the evolved gases discovered via the TGA-FTIR analysis were largely impacted by the size of the initial biomass. At lower pyrolysis rates, like the one used in this study, the mass loss for samples just under 1 mm was less than that of samples less than 27 μm^21^. Suggesting that more mass was maintained in this process to form carbonaceous materials. However, samples closer to 1 mm as compared to 100 μm release more carbon dioxide during the pyrolysis process^21^. As compared to the original 1 cm sized samples, the milled PHs may have released more hydrogen and carbon monoxide but less carbon dioxide^22^. Beneficially, the reduced size of the biomass required less activation energy to pyrolyze the samples^21,48^. A cost to benefit ratio would be required for implementation of this material on a larger scale. The use of this material is far more thermally stable than traditional natural fillers and can be used in a larger number of high temperature applications (automotive and electronics industries for example). Many food or beverage industry wastes degrade at temperatures less than 200 °C^3,28^. As well this material could act as a natural colourant to replace the existing carbon black material which would produce its own volatile materials.

#### Raman spectroscopy

**Figure 7:**
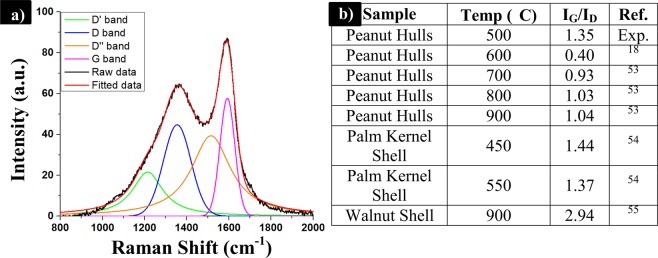
(**a**) Raman spectra for PH 500 °C biocarbon and (**b**) comparison of I_G_/I_D_ ratios with other nut shells reported in literature.

Deconvolution of the Raman spectrum to determine the D and G bands of the samples was performed from 800 to 2000 cm^−1^. A total of four peaks were fitted to the curve, as shown in Fig. 7. The D and G bands experienced peak maxima at 1352 cm^−1^ and 1588 cm^−1^. Two additional peaks corresponding to D’ and D” were fitted at peaks located near 1200 cm^−1^ and 1510 cm^−1^, respectfully. These types of peak have been associated with disordered carbon where the D’ peak has been specifically attributed to a sp^3^ phase of amorphous carbons^49,50^. The D” peak is associated with the phonon density of the different states present in finite size graphitic crystals. Alternatively, the D” peak may be attributed to vibrations of the C-H groups contained within hydrogenated carbons^50^. On the contrary, Ferrari and Robertson have suggested that the D’ and D” peaks are resultant of the sum and differences between stretching of C=C and wagging of C-H contained in the trans-polyacetylene groups. The trans-polyacetylene groups are formed from chains of alternating hydrogen to carbon bonds as well as sp^2^ carbons. Furthermore, the previously mentioned chains are contained within the nanocrystalline diamond structures and result from sp^3^ carbons^51^. The presence of these functional groups has been confirmed by FTIR. The D’ and D” peaks were also identified on the spectrum of nano-graphite. The nano-graphite was prepared via ball milling of the big graphite flakes. Therefore, this suggests the D’ and D” bands identified in peanut biocarbon may not be from the remaining functional groups nor the sp^3^ carbons, but instead are from finite sized crystallites and the subsequent increase in biocarbon defects. More study is needed to establish the exact cause of these peaks.

The I_G_/I_D_ peak ratio was substantially greater than for other PH biocarbon samples^17^ with a similar pyrolysis temperature. However, from literature it appears that this value can increase with increasing pyrolysis temperature, and also varies with biomass type. The very small ratios suggest that the graphitic ordering of the samples is limited^17^. The PHs have also been compared to walnut shell^52^ and palm kernel shells^53^ and it was determined that the PHs possessed very similar results to that of palm kernel shells. It is important to note that these values are strongly impacted by the pyrolysis conditions.

Further analysis of the Raman spectra over the range of 2000–3400 cm^−1^ was completed to look for characteristic structures. The graph is displayed in Fig. S2 of the Supplementary Information. There is a broad peak spanning 2600 to 2900 cm^−1^. In literature, carbon-based materials have shown peaks at 2700 cm^−1^ which are associated with single graphite crystals^54^. This correlates with the greater intensity obtained for the graphitic peak versus the disorder peak in the lower range on the Raman spectra.

#### Conductivity

The electrical conductivity of PH biocarbon, displayed in Table 8, was determined to be of the same magnitude as that of softwood^34^ and coffee chaff^18^. There are several factors that determine the electrical conductivity of a sample, including pyrolysis temperature, particle size, crystalline structure of biocarbon and surface elements^34^, as well as the carbonization of biocarbon^55^. The pyrolysis temperature in this experiment was only 500 °C which may have led to a few aromatic structures being present, and thus fewer electrons available to conduct electricity^34^. Likewise, the pyrolysis temperature affects the carbonization of the biocarbon such that a few percent increase in carbon content could lead to magnitudes of difference in electrical conductivity^55^. The electrical conductivity of the samples is studied to determine suitable applications for the biocarbon. For example, in agriculture applications where biocarbon is used as a soil absorbent, the electrical conductivity relates to the salinity of the biocarbon^56^ and its ability to interact with other compounds in the soil.

**Table 8:** Thermal and electrical properties for biocarbon.

Sample	Thermal Conductivity (mW m^−1^ K^−1^)	Thermal Diffusivity (10^−2^ mm^2^ s^−1^)	Specific Heat (MJ m^−3^ K^−1^)	Electrical Conductivity (mS m^−1^)
500 °C Peanut hull (PH) Biocarbon	103 ± 1.73	4.15 ± 1.76	2.47 ± 0.0737	3.88 ± 0.147

The thermal properties, including conductivity, diffusivity and specific heat, are shown in Table 8. The thermal properties of PH biocarbon were similar to those of softwood samples pyrolyzed at the same temperature^34^. Thermal properties of biocarbon are important to determine how heat is transferred and to determine industrial applications for the material. Usually, heat is transferred through biocarbon in the form of radiative energy (electrons and photons) through the pores^57^. A lower thermal conductivity would be beneficial for insulating applications or where the final product needs to withstand higher temperatures.

#### Surface area analysis

BET surface area for samples ball milled and pyrolyzed at 500 °C was 117.3 m^2^/g. Wood chip biochar was also subjected to slow pyrolysis at the same temperature as PHs and followed the exact same ball milling procedure. The surface area for this woody biomass was very similar at 103.5 m^2^/g^34^. However, other woody biomass such as soy beans and coffee chaff subjected to pyrolysis and milled at the same conditions only produced surface areas of 10.7 m_2_/g and 11.7 m^2^/g, respectively. As noted from the SEM images, the biocarbon generated from PHs possessed sheet like structures offering more exposed surface area.

#### X-ray diffraction analysis

**Figure 8:**
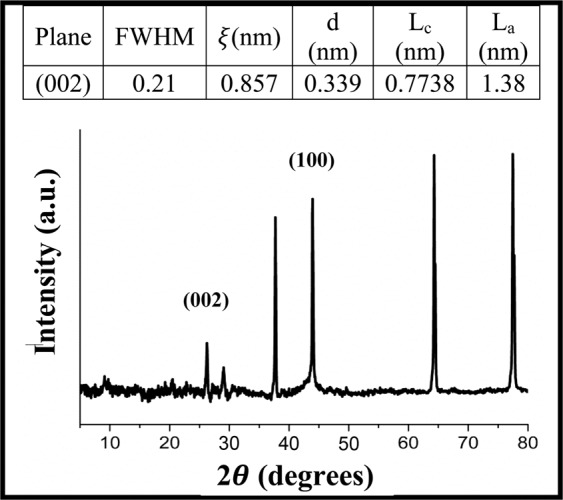
XRD analysis for PH biocarbon at 500 °C.

The x-ray diffraction (XRD) analysis examined the crystalline structure with the PH biocarbon sample and can be found in Fig. 8. The graphite content can be identified by the peak (002) at 2 *θ* = 26.3° ^58^. The graphitic content was also confirmed in Raman spectra. Interestingly, PH biocarbon is able to obtain this structure without the addition of catalysts whereas *Miscanthus* grass requires the addition of cobalt or iron to achieve similar peaks^58^. The domain size (*ξ*) for the (002) structure was found to be 0.86 nm. Another plane (100) was located at 2 *θ* = 43.9° was associated with graphitic content^59^. Similar peaks have been found in PH biocarbon^60^. The combination of the two planes, (100) and (002) can be used to calculate the stacking height (L_c_) and lateral crystallite size (L_a_). The L_c_ value was very similar to other pyrolyzed lignocellulose in literature^19^. Other major peaks were located at 2 $\theta$ = 29.0°, 37.7°, 67.68° and 77.5°. The results obtained from XRD analysis further support the findings in Raman and may be the result of the unique and highly organized (in appearance) of the morphology via SEM analysis

### Composites

#### Mechanical performance of composites

Composites were produced with an engineering thermoplastic, PTT. An important reason for blending with this polymer was to increase the bio-content from 35 wt.% for the neat polymer to 48 wt.% (20 wt.% from filler and 35% of the 80 wt.% PTT) (Table 9). Biocarbon is a cost effective and sustainable filler that could offer alternatives to other fillers such as talc, glass fiber or petroleum based carbon black^61,62^. Biocarbon also offers improved thermal stability over that of traditional natural fibers, as noted from the TGA analysis. The mechanical properties of the composites can be found in Table 9.

**Table 9:** Mechanical properties of neat PTT and peanut hull biocarbon composites.

Materials	Impact Strength (J/m)	Biobased content (wt.%)
Neat PTT	33.33 (0.96)	35
80:20 (PTT: Peanut hull Biocarbon) (500 ^o^C)	16.97 (1.11)	48

**Figure 9:**
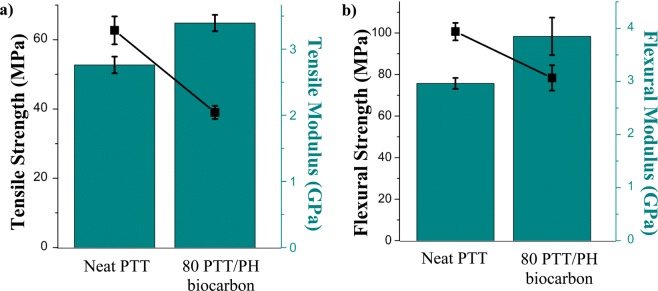
(**a**) tensile properties and (**b**) flexural properties for neat PTT and 500 °C PH biocarbon composite samples.

**Figure 10:**
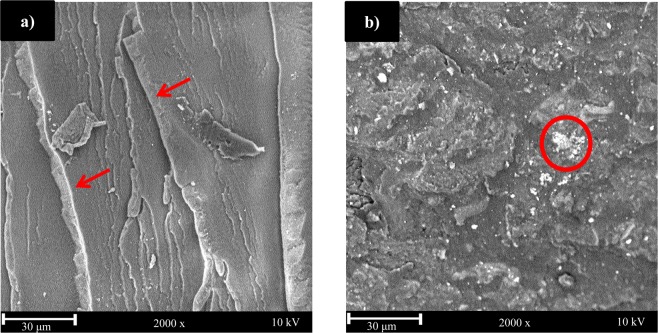
SEM images of (**a**) neat PTT and (**b**) 80wt.% PTT with 20 wt.% 500** °**C PH biocarbon.

There was decrease in the tensile strength and flexural strength in the composites by 60% and 28%, respectively (Fig. 9). However, there was an increase in the tensile and flexural modulus by 137% and 140%, respectively. The impact strength also decreased from about 33 to 17 J/m with the addition of 20 wt.% biocarbon. Other biocomposites containing 20 wt.% biocarbon and 80 wt.% PTT were also found to have decreased impact strength due to the increased brittleness^63^, which was confirmed by SEM (Fig. 10b). The decrease in mechanical performance of the biocarbon can be attributed to the particle size and surface functionalities of the biocarbon, as well as to the interfacial adhesion between the matrix and filler^64,65^. The biocarbon used in this experiment was relatively large as compared to mineral fillers^63^ or other reported uses of biocarbon in composites^36^. The biocarbon in this work was only ball milled for 1 hr. In works completed by Codou *et al*., samples of biocarbon were milled for 24 hr, and thus resulted in better strength properties^37^. Longer duration of ball milling and possible surface modifications for the PH biocarbon or another method of size reduction could be done in future works to improve the mechanical performance of the biocomposites.

### Morphological analysis

Scanning electron micrographs were taken of the impact surface of neat PTT and PH biocarbon samples. The ridges on the neat PTT samples, noted by the red arrows on Fig. 10a, are associated with a brittle fracture surface. The differences in the fracture surface confirm the increased brittleness of the samples containing biocarbon, as also found in works with similar materials^63^. The PH biocarbon samples do show some flocculation (highlighted in red circles on Fig. 10b) of particles which may have impacted the mechanical performance. According to literature, higher filler contents (i.e. 20–30 wt.%) are more likely to agglomerate, and also finer material has increased tendency to agglomerate^66^. This was consistent with the findings of this work.

### Benefits and feasibility of biocarbon

Biocarbon produced from PHs, is more sustainable in comparison to traditional inorganic fillers^64^. There is also a reduction in density of PH biocarbon as compared to traditional fillers, suggesting their replacements with a biobased and sustainable alternative^64^. The composites may be used in the automotive sector and electronic industries where light-weighting and improved sustainability are essential for implementation of new materials. Moreover, the biocarbon has improved thermal stability over that of other traditional natural fillers^3^. Such benefits of this material, in addition to the vast quantiles produced each year show promise in sustainable applications.

## Conclusions

Peanut hulls are the outermost shell of the peanut and can be obtained as a low cost by-product from the food processing industry. To date, there are some suggested uses of peanut hulls for bioenergy, but most PHs are spread back on the field or discarded as waste. There is a substantial desire to valorize these materials to contribute to a circular economy for sustainable product development. Therefore, this work features complete morphological, chemical and thermal analysis of the material and one suggested application. The PHs possessed a unique surface morphology, as noted through SEM analysis, relatively low ash content and have exceptional thermal stability compared to traditional natural fillers. The sheet-like surface morphology was found to have higher graphitic carbon content than disordered carbon based on Raman analysis. And this was further supported by XRD analysis where the (002) and (100) planes, (those associated with graphitic content), were found. This suggested the materials use for sustainable composite applications. To further improve the thermal stability of the material for high temperature biocomposite applications, the PHs were subject to thermochemical conversion (pyrolysis). Samples were heated to 500 °C in an inert atmosphere. After the pyrolysis, the resulting biocarbon was examined for its chemical structure, conductivity and morphology. Raman spectroscopy determined that the samples contained higher graphitic content than disordered carbon content. The electrical conductivity was relatively low, suggesting uses in anti-static applications. The surface morphology of the samples was maintained after the pyrolysis process. The PH biocarbon was combined at 20wt.% with 80wt.% poly(trimethylene terephthalate) (PTT). The resulting composites improved the renewability content to 48% and experienced increases in both the flexural and tensile moduli by at least 130%. The successful fabrication of PH biocarbon composites is just one of the potential, sustainable and value-added uses which peanut hulls offer.

## Supplementary information

Supplementary Information.
