# Supplementary material for: Biocarbon from peanut hulls and their green composites with biobased poly(trimethylene terephthalate) (PTT)
Source: Sci Rep. 2020 Feb 24;10:3310. doi: 10.1038/s41598-020-59582-3 (PMC7039894; doi:10.1038/s41598-020-59582-3)
Supplement: Supplementary file 1 — Supplementary Information. [file 41598_2020_59582_MOESM1_ESM.docx]

**Biocarbon from peanut hulls and their green composites with biobased poly(trimethylene terephthalate) (PTT)**

**Maisyn Picard^1,2^, Suman Thakur^1^, Manjusri Misra^1,2,*^, Deborah F. Mielewski^3^,**

**Amar K. Mohanty^1,2,*^**

*^1^ Bioproducts Discovery and Development Centre, Department of Plant Agriculture, University of Guelph, Crop Science Building, 50 Stone Road East, Guelph, Canada*

*^2^ School of Engineering, University of Guelph, Thornbrough Building, 50 Stone Road East, Guelph, Canada*

*^3^Research and Innovation Center, Ford Motor Company, Dearborn, MI 48124, USA*

*^*^Corresponding authors: mmisra@uoguelph.ca, mohanty@uoguelph.ca*





**Wavenumber (cm^-1^)**

**Absorbance (a.u.)**

Figure S1: Gram-Schmidt plot for peanut hulls.





**Raman Shift (cm^-1^)**

**Intensity (a.u.)**

Figure S2: Raman spectra from 2000-3400 cm^-1^ for peanut hull biocarbon at 500 ºC.
